# A Framework for Femtech: Guiding Principles for Developing Digital Reproductive Health Tools in the United States

**DOI:** 10.2196/36338

**Published:** 2022-04-28

**Authors:** Tamar Krishnamurti, Mehret Birru Talabi, Lisa S Callegari, Traci M Kazmerski, Sonya Borrero

**Affiliations:** 1 Division of General Internal Medicine University of Pittsburgh Pittsburgh, PA United States; 2 Center for Innovative Research on Gender Health Equity University of Pittsburgh Pittsburgh, PA United States; 3 Division of Rheumatology and Clinical Immunology University of Pittsburgh Pittsburgh, PA United States; 4 Department of Obstetrics and Gynecology University of Washington School of Medicine Seattle, WA United States; 5 Health Services Research and Development Veterans Affairs Puget Sound Health Care System Seattle, WA United States; 6 Department of Pediatrics University of Pittsburgh Pittsburgh, PA United States; 7 Center for Health Research and Promotion Veterans Affairs Pittsburgh Healthcare System Pittsburgh, PA United States

**Keywords:** United States, North America, femtech, mHealth, health equity, pregnancy, women's health, preterm birth, contraception, family planning, reproductive care, sterilization, cystic fibrosis, rheumatic disease, eHealth, mobile health, reproductive health, digital health, health technology, health outcomes

## Abstract

The United States has abysmal reproductive health indices that, in part, reflect stark inequities experienced by people of color and those with preexisting medical conditions. The growth of “femtech,” or technology-based solutions to women’s health issues, in the public and private sectors is promising, yet these solutions are often geared toward health-literate, socioeconomically privileged, and/or relatively healthy white cis-women. In this viewpoint, we propose a set of guiding principles for building technologies that proactively identify and address these critical gaps in health care for people from socially and economically marginalized populations that are capable of pregnancy, as well as people with serious chronic medical conditions. These guiding principles require that such technologies: (1) include community stakeholders in the design, development, and deployment of the technology; (2) are grounded in person-centered frameworks; and (3) address health disparities as a strategy to advance health equity and improve health outcomes.

## Background

Black birthing people living in the United States are four times more likely to die during the peripartum period than those who are white, regardless of income, education, or severity of complications [1,2]. Black Americans, Indigenous Americans, and Latinas residing in the United States all experience significantly higher rates of preterm delivery than those who are white [3]. Drivers of reproductive health disparities, including personally mediated and institutional racism, manifest in myriad ways, from housing inequities and unequal access to safe environments to education, opportunity, and health care [4,5]. In addition, individuals with serious chronic medical conditions—particularly those from socially marginalized groups—may be less likely than healthy people to receive critical health interventions, including access to high-quality contraceptives and other reproductive health care [6] and prenatal services [7]. Research by our team members identified critical gaps in current reproductive health care delivery models, especially for people from socially and economically marginalized populations, those with serious chronic medical conditions, and those with intersectional identities that are subject to both social and medical marginalization. These gaps in care undermine proactive approaches to identify reproductive health needs and thereby address preventable adverse outcomes.

A host of new software and digital tools have emerged from the private and public sectors to address women’s reproductive health needs. Colloquially referred to as “femtech,” these technologies range from menstrual cycle–tracking apps to medical devices for pelvic floor strengthening. In the private sector, femtech is expected to constitute a US $50 billion industry by 2025 [8]. While femtech represents a promising opportunity to address existing gaps in women’s health care, the intended recipients of femtech innovations largely appear to be healthy, affluent, white, cis women. In the current model, an opportunity is missed to engage populations who have been historically underserved and bear the largest burden of poor pregnancy and perinatal outcomes.

This viewpoint outlines three key principles for developing scientifically grounded digital tools that bridge critical gaps in women’s health care among individuals who have been subjected to social, economic, and medical marginalization. We illustrate the implementation of these principles with examples taken from our own development of tools that span reproductive health transitions or decisions, including family planning, pregnancy, and sterilization. These principles are equally applicable to the development of research and commercial tools, and offer a set of guidelines to broaden femtech to more equitably address women’s health needs.

Here, “women’s health” refers to the area of research dedicated to the treatment and diagnosis of diseases and conditions that affect those with female physiology and, in this instance, female reproductive health. As such, we use the terms “woman” and “women” throughout this paper. However, we acknowledge that these are gendered terms and the intended users of femtech do not necessarily identify as women. An important future consideration for our framework and the femtech field more broadly will be enhancing gender inclusivity in both the language and methods we use.

## Our Principles

### Collaborative Development Strategy

Our collaborative of university-based researchers first joined to garner intellectual support and collaboration around our shared interests across diverse medical and scientific subspecialties. We developed the following shared principles to formalize guidance for our own reproductive digital health tool designs. Lessons learned were obtained through trial and error.

### Create Interdisciplinary Stakeholder–Inclusive Teams

Cross-disciplinary collaboration is required to ensure that any reproductive health tool is comprehensive and accurate. Clinical experts offer scientifically grounded content and appropriate clinical actions. Social scientists explain human behavior and the social structures that constrain or shape behaviors. Visual designers capture the clinical and social scientists’ input through a user interface. Technical experts realize the teams’ vision into a functional and scalable tool.

However, academic and technical partnerships are not sufficient. Echoing tenets of user-centered design, those who are building tools must have a solid understanding of who the users will be and how they will interact with the tool [9]. We take this a step further by suggesting that representatives of all stakeholder groups, particularly patient stakeholders, must be included in the entire process from design to implementation. Patients, for example, should serve not only as testers but as active participants in shaping the conceptual and pragmatic underpinnings of the project, and are thus a critical part of an interdisciplinary team. The specific stakeholders’ needs and the methods in which they are engaged in the development process will inevitably differ from tool to tool and by the specific health context being addressed.

### Facilitate a Person-Centered Approach

Over the past 20 years, person-centeredness has increasingly been recognized as an indicator of high-quality health care [10]. While patients are now acknowledged as active partners in the clinical decision-making process [11], a power asymmetry intrinsically exists between clinicians and patients [12]. Clinicians may control patients’ access to reproductive services by selecting which procedures they conduct or which referrals they order; they are thereby positioned to support or undermine an individual’s ability to actualize their reproductive goals and preferences [13]. Furthermore, women experiencing social marginalization may have different decision-making constraints, values, or choice architecture, leading to different, but equally valid, decisions [14].

It is an ethical imperative that individuals who are the desired users of these tools have the power to be active participants in their health care decisions and that their right to make their own reproductive decisions is honored, regardless of the context. Thus, tools must aim to facilitate meaningful understanding of the risks, benefits, and uncertainties associated with various reproductive health decisions, helping users to clarify their personal preferences as they make their choices. Some of our tools aim to help patients make decisions that are aligned with their preferences and values. Some tools offer personalized feedback on patients’ specific health needs. Other tools are designed to support shared decision-making between users and their clinicians. All of our tools serve the purpose of supporting a patient’s autonomy and self-efficacy to achieve their desired reproductive health goals.

The success of a digital reproductive health tool must, in part, be judged on its outcomes. We believe that these outcomes, much like the approach to tool design and functionality, must be person-centered. In a clinical setting, this includes ensuring that changes in clinical as well as patient-reported outcomes are aligned with the values of the patients themselves. While the success of femtech is usually evaluated on widespread adoption or a revenue stream if commercialized, our mission is to build and implement tools that improve health care experiences and support health outcomes that are desirable to the patient. Such outcomes may range from interpersonal (eg, feeling respected) to psychosocial (eg, decreased decisional conflict) to clinical (eg, less disparity in preterm births). The key is that these outcomes must be grounded in what patients value as individual *people* and not what affects the fiscal or other priorities of the health care establishment serving them. Ideally, tools that can improve reproductive health in a person-centered manner will also be widely adopted and consequently improve the efficiency and quality of health services.

### Advance Reproductive Health Equity

To advance reproductive health equity and address the needs of populations whose reproductive health and well-being have been harmed or neglected by our current societal structures, femtech work must have a race-, class-, and gender-conscious approach from its inception. We suggest specific frameworks that guide our development process, including Intersectionality Theory [15], Critical Race Theory [16], the R4P Framework [17], and the Reproductive Justice Framework [18]. These specific frameworks are grounded in racial justice and US civil rights; nevertheless, considerations of intersectionality and the larger message of these frameworks are globally relevant [19]. Moreover, we urge constant self-reflection on each step of the process [20].

## Practical Application of These Principles

Here, we offer additional details about our collaborative’s suite of tools, each of which were built using our guiding principles to address a unique decision-making period in the reproductive journey: reproductive care for nonpregnant people, pregnancy, and sterilization. Table 1 outlines how to incorporate these guiding principles into the different phases of tool development. As we describe each tool, we offer practical illustrations.

**Table 1:** Ways to incorporate principles into phases of femtech development.

Phase of development	Principles		
	Interdisciplinary stakeholder–inclusive teams	Person-centered approach	Advancing reproductive health equity
Conceptualization and content development	Conduct semistructured qualitative work (one-on-one interviews or focus groups) with women, health care providers (including subspecialty providers), and other expert stakeholders	Structure interview guides around evidence-based best practices to identify gaps in knowledge, and understand experiences and preferences related to reproductive care	Incorporate historical and theoretical frameworks in conceptualizing the tool and its content to ensure an equity lens from the start of any work
Design implementation	Review content and functionality iteratively with members of key stakeholder groups such as patients, medical experts, human-computer interaction specialists, bioethicists, social scientists, and relevant community organization leaders (eg, reproductive justice advocacy groups, church leaders, women’s shelters, doulas)	Design features and content to incorporate clinical best practices, yet focus on users’ informational needs and personal values	Structure advisory or expert panels to include content and lived-experience experts; seek diverse perspectives within each category of stakeholder
Testing	Prioritize patient and other stakeholder goals for the tool	Plan acceptability metrics around patient-centered/patient-defined outcomes	Power trials to identify differences in outcomes for diverse patient populations based on preplanned equity-driven hypotheses

## Reproductive Care for Nonpregnant People With MyPath

National guidelines recommend that patient-centered reproductive services be routinely provided in preventive care settings to help individuals optimize health and well-being prior to desired pregnancies and to prevent unwanted pregnancy and births [21]. Despite these recommendations, contraception and abortion counseling is frequently absent from preventive health encounters [22,23] and, when it does occur, often fails to prioritize individual preferences, values, and goals [24,25]. Individuals in socially marginalized groups, including people of color and those with low incomes, perceive a lower quality of care [26] and report experiences of discrimination and pressure to use contraception, to choose certain methods, or to limit family size [24,27]. Our understanding of what constitutes high-quality reproductive care has shifted in recent years, driven by advancements in research and social movements [28,29]. This evolution has included moving from approaches focused on strict pregnancy planning and reducing individual risk behaviors toward approaches that include assessment of individuals’ goals and needs, acknowledge the structural factors (eg, racism and poverty) underlying poor reproductive health outcomes, and prioritize reproductive autonomy [28]. The benefits of person-centered counseling strategies such as shared decision-making, which acknowledge the complexity of contraceptive preferences and help individuals match those preferences with their choices, are increasingly being recognized [27]. At the same time, the potential harms of directive counseling approaches, which advocate for one contraceptive method or group of methods over others, have also been brought to light, particularly in communities that face historic and ongoing reproductive oppression in the United States [30,31].

**Figure 1:**
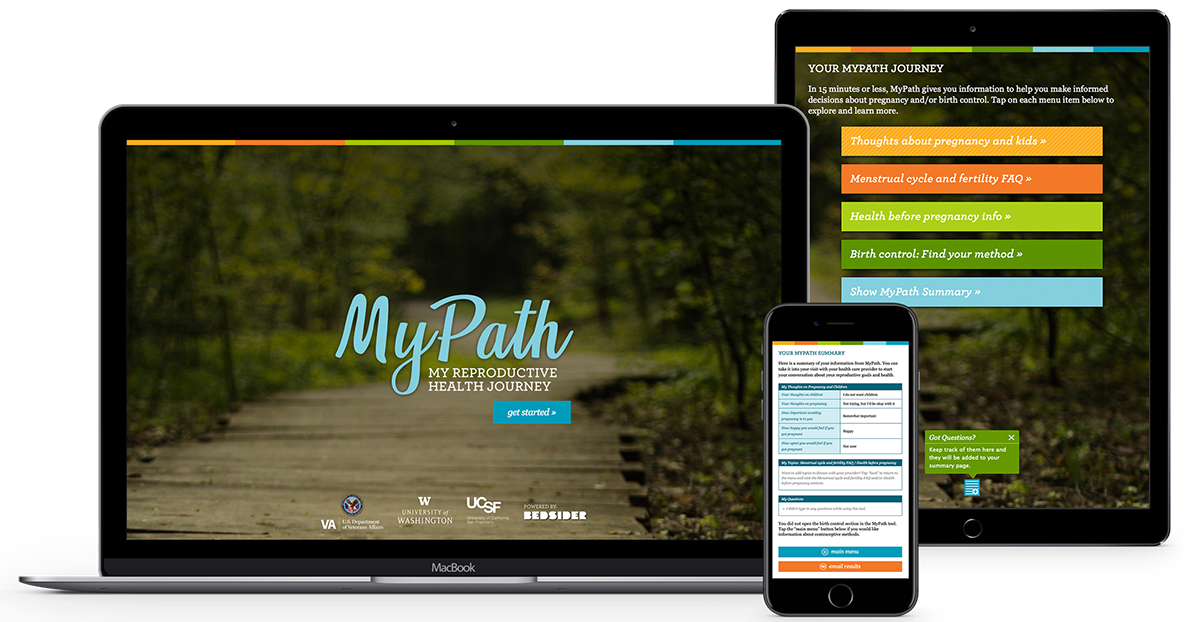
MyPath online decision support tool.

Figure 1 shows MyPath, a patient-facing web-based reproductive decision support tool that we developed to promote high-quality, person-centered discussions about reproductive needs in the Veterans Health Administration (VA). Veterans capable of pregnancy who use VA Health Care services are a highly diverse population, with nearly half identifying as racial or ethnic minoritized groups [32], and face elevated risks of adverse pregnancy and birth outcomes compared to their civilian counterparts due to a higher prevalence of medical, mental health, and psychosocial factors [33]. Drawing on our first principle (*Principle 1: Interdisciplinary stakeholder–inclusive teams*), MyPath development was informed by formative qualitative work to understand veterans’ preferences and needs [25] and followed user-centered design principles, guided by patient, provider, and scientific expert input [34]. Designed to be used prior to primary care visits, MyPath empowers patients by helping them clarify and share their reproductive goals with their primary care providers, augmenting their knowledge, and building self-efficacy in communicating about their reproductive needs (*Principle 2: Facilitating a patient-centered approach*). By centering individuals’ preferences, needs, and goals, MyPath aims to safeguard reproductive autonomy and address inequities in reproductive health care related to poor provider-patient communication (*Principle 3: Advancing reproductive health equity*).

In a nonrandomized pilot of the final prototype (control group, n=28; intervention group, n=30), we found that a greater proportion of intervention participants reported having discussions about their reproductive needs in the visit compared to controls (93% vs 68%; *P*=.02) [34]. A multisite hybrid effectiveness-implementation randomized controlled trial is currently ongoing to evaluate the impact of the text message–delivered MyPath on various person-centered and clinical outcomes, with the goal of ultimately scaling the intervention nationally within VA if found to be effective.

## Pregnancy Support With MHP MyHealthyPregnancy

Preterm births, those that occur prior to 37 weeks of gestation, are the leading direct cause of neonatal mortality and morbidity [35]. More than 1 in 9 births in the United States are preterm [36], and Black women are roughly 60% more likely than white women to have a baby born prematurely. The causes of preterm birth are complex, with a wide range of risk factors identified in the literature, some of which may never be identified by the provider during routine care, such as intimate partner violence (IPV) [37], depression [38], or chronic toxic stress [39]. Even when risk factors are identifiable and addressable, physicians may find it difficult to communicate recommendations effectively enough to support risk mitigation [40]. Physician-patient communication challenges are particularly prevalent for Black patients, who, compared to non-Hispanic white patients, report that they are unlikely to ask questions of their physicians and that their physicians are less likely to listen when they do [41]. Even for those physicians that are aware of patients’ particular informational needs, time constraints may prevent more in-depth discussion. Addressing these adverse events requires both accurate identification of an individual’s risk factors and actionable communication of those risk factors to women and their health care providers.

**Figure 2:**
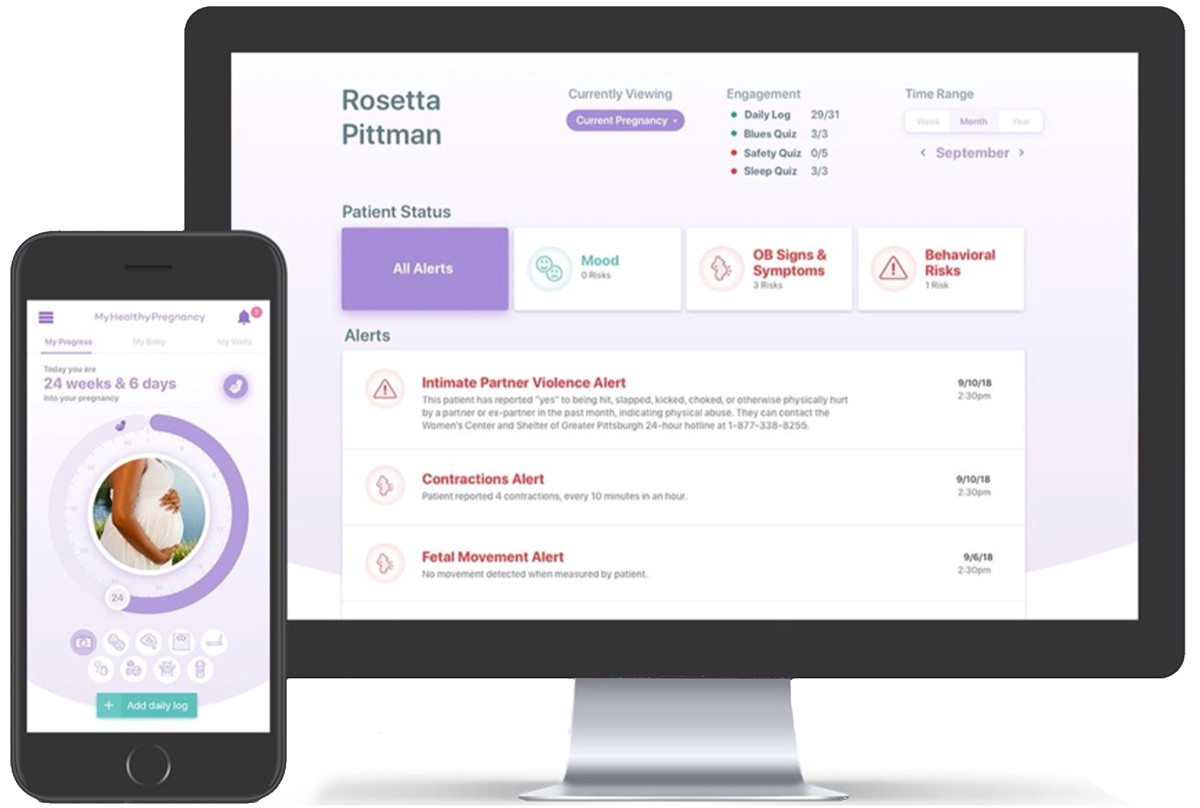
The MHP MyHealthyPregnancy smartphone app (left) and provider portal (right).

The MHP MyHealthyPregnancy mobile health platform shown in Figure 2 comprises a patient-facing smartphone app and an electronic health record–integrated provider portal. MHP MyHealthyPregnancy uses real-time data collection from pregnant people via the app [42] and applies statistical machine learning algorithms [43] to those data to identify modifiable precursor risks to preterm birth between routine prenatal care visits. As risk factors are identified, they are communicated to health care providers in real time through a provider portal. The app, in turn, offers patients sensitive, respectful, and actionable risk minimization strategies and resources.

MHP MyHealthyPregnancy content and flow were initially drafted by an interdisciplinary team (*Principle 1: Interdisciplinary stakeholder–inclusive teams*), including medical experts in the field of maternal-fetal medicine as well as human-computer interaction specialists and community informants (eg, church leaders, nonprofit organizations, women’s shelters, doula groups). All experts had a voice in determining how to assess and intervene on the specific risk factors that fell within their own area of expertise. Next, we worked directly with pregnant and recently pregnant women, purposively recruiting a socioeconomically and racially diverse group, to identify their current beliefs, values, and constraints with respect to using such a pregnancy tool. The features we incorporated into the app were designed iteratively with the women we interviewed to specifically address expert-identified risks while remaining sensitive to the users’ needs (*Principle 2: Facilitating a patient-centered approach*). For example, our medical experts highlighted a need to provide education about symptomatic bleeding; yet, many of the peripartum women we spoke with reported confusion between spotting, miscarrying, and menstruation. As such, the content we produced had to be acceptable and understandable to both types of stakeholders. Our app-based solution was a daily symptom assessment with feedback on the need for immediate medical care when appropriate [42].

MHP MyHealthyPregnancy was tested in a quality-improvement initiative in UPMC (formerly known as the University of Pittsburg Medical Center) health care system from September 23, 2019, to September 1, 2021, with over 5600 English-speaking pregnant people; evaluation analyses are ongoing. Of those offered MHP MyHealthyPregnancy by their provider, 81.5% of patients initiated use of the tool. Initial findings show promising results for MHP MyHealthyPregnancy for filling gaps in clinical risk detection and, ultimately, prevention. In an analysis of 959 patients who used the app for reporting IPV, 100% of those reporting a current physical risk of IPV had no mention of IPV in their medical charts, despite treatment for injuries that should have prompted in-person screening administration [44]. MHP MyHealthyPregnancy was similarly able to successfully implement screening for preeclampsia risk factors among 2563 patients, with more than half of those app users who met the highest preeclampsia risk criteria reporting no preeclampsia prophylactic recommendation from their provider [40]. Patients with baseline reports of certain clinical risk factors such as a history of depression or prior preeclampsia have engaged with the app in significantly higher numbers than those without a baseline history of pregnancy risk factors. To address challenges identified with provider bandwidth in responding to notifications in the provider portal, we built in an alert to be directly sent to a dedicated clinical care team consisting of nursing staff. This approach allowed for an increased response to app-based detection of depression, facilitating connection to behavioral health services.

For any digital tool that addresses reproductive health needs, a one-size-fits-all approach will likely fail. For example, in creating a Spanish-language version of MyHealthyPregnancy (*MHP Embarazo Saludable)*, our first step was not a straight translation of the existing content into Spanish, but rather to reframe and then translate the content to address the unique needs of Latinas not born in the United States, who may be navigating a system and approach to pregnancy health care that differ substantially from their prior experience. For example, many Latinas we spoke with who were recent immigrants discussed their concerns about social isolation, particularly given the supportive role that female relatives traditionally play in transitioning to motherhood [45]. Therefore, for the Latina population, explicitly facilitating a connection to relevant social supports (eg, Spanish-language doulas) based on when a need is identified through MHP MyHealthyPregnancy is a person-centered approach that may affect equitable health outcomes, as identified by the patients themselves (*Principle 3: Advancing reproductive health equity*). A multisite pilot study, funded by the Centers for Disease Control and Prevention, is currently examining the effectiveness of person-centered approaches for psychosocial screening and support with both Spanish and English versions of the tool at two demographically diverse sites.

## Sterilization Decision-Making With MyDecision

Female sterilization, which is currently the most commonly used contraceptive method in the United States, is disproportionately used by those with social disadvantages, including those with low incomes, with public or no insurance, with lower educational levels, and from racial/ethnic minority groups [46,47]. The reasons for this are complex and exist within a broader social context that includes a history of sterilization abuses as well as ongoing devaluation of socially marginalized women’s reproduction. Research findings illuminate various tensions and potentially countervailing forces that exist in the decision to use sterilization as a contraceptive method and the ability to execute such decisions. For example, women report that sterilization decisions are largely driven by personal preferences and prior reproductive experiences [48,49]. However, among those who have undergone the procedure, there is a significant level of misunderstanding about sterilization (eg, the permanence of the procedure) and alternative contraception methods, as well as a relatively high prevalence of poststerilization regret [50], suggesting suboptimal counseling and decision-making. Additionally, although rates of sterilization are higher among those with low incomes and people of color, many from these groups also report restricted access to the procedure due to provider reluctance to perform it for various reasons (eg, concern that the patient will ultimately regret the decision) as well as barriers posed by stringent Medicaid sterilization consent policies, and these issues are perceived as undermining their reproductive autonomy [51].

**Figure 3:**
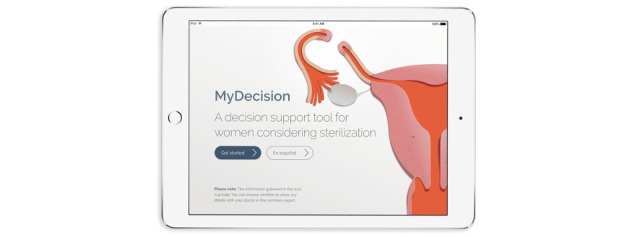
The MyDecision web-based tool.

The content of the MyDecision tool, as shown in Figure 3, was informed by foundational, in-depth interviews with racially diverse, low-income women who had ever considered tubal sterilization, as well as with health care providers who perform female sterilization to understand informational and decision support preferences and needs. Several of the women participants were also included on a Steering Committee that helped guide the design and structure of the tool throughout the development process. As the history of sterilization and its consent among low-income women have been socially and ethically fraught, Steering Committee members also included representation from women of color and reproductive justice advocacy groups, the American College of Obstetricians and Gynecologists (ACOG) Ethics Committee, and the ACOG Committee on Health Care for Underserved Women. To ensure that the voices of our “patient” participants were heard, we held separate meetings with them in addition to their participation in the larger Steering Committee meetings (*Principle 1: Interdisciplinary stakeholder–inclusive teams*). Once a prototype of the tool was approved by the Steering Committee, cognitive testing with end users (ie, women with low incomes and considering tubal sterilization) was performed to assess comprehensibility and usability of the tool and make subsequent refinements, consistent with human-centered design principles.

Dismantling the potential for sterilization abuses will require grappling with the complicated social and political forces that stratify reproductive value. However, the MyDecision patient-facing, web-based decision aid seeks to address a more feasible, yet necessary, objective: to empower low-income English- and Spanish-speaking women who are contemplating undergoing a sterilization procedure by providing them with unbiased, relevant information, and a process with which to *independently* engage in informed and value-concordant decision-making and communicate their preferences to their health care providers. The overarching goal of the tool is to better support low-income women’s bodily and reproductive autonomy and help them achieve their reproductive goals (*Principle 2: Facilitating a patient-centered approach* and *Principle 3: Advancing reproductive health equity*).

The MyDecision tool is currently being tested in a National Institutes of Health–funded multisite randomized controlled trial to determine its efficacy in improving decision quality. If found to improve sterilization decision quality and help ensure informed and voluntary consent, MyDecision could potentially offer a scalable and evidence-based alternative to the current problematic Medicaid sterilization consent process.

## Tools to Address Considerations Related to Chronic Medical Conditions With MyVoice:Rheum and MyVoice:CF

People who have chronic medical conditions may have reproductive health concerns that are both general and disease-specific. Our team developed two patient-directed tools to support family planning care for women with rheumatic and musculoskeletal diseases (RMDs) and cystic fibrosis (CF); these conditions are independently associated with pregnancy-related concerns, including potentially higher rates of maternal morbidity and mortality and/or adverse neonatal outcomes [52]. Approximately 7 million women in the United States have RMDs, including autoimmune and connective tissue diseases, and many of these diseases are diagnosed during reproductive age [53]. Owing to the advent of new and highly effective medications targeting the underlying defect in CF, people with CF are anticipating longer, healthier lives, and many are considering and pursuing pregnancy [54]. Among women with RMDs and CF, family planning care is therefore essential for supporting informed decision-making about: (1) the benefits and risks of pregnancy, (2) medication safety in the context of pregnancy and lactation, (3) pregnancy intentions and preferred timing for pregnancy and/or parenthood, and (4) selection of safe and acceptable contraceptive methods [55,56].

However, family planning discussions rarely occur in either the RMD or CF subspecialty care contexts [57,58]. Many subspecialty clinicians are inadequately trained to provide consistent, accurate, or high-quality family planning care. Obstetricians and primary care providers often lack adequate disease-related knowledge to provide family planning care that comprehensively addresses patients’ information needs [59]. Furthermore, clinicians may have social biases that lead them to counsel patients about their reproductive options differently with consideration of their social and economic backgrounds in addition to their health [60].

**Figure 4:**
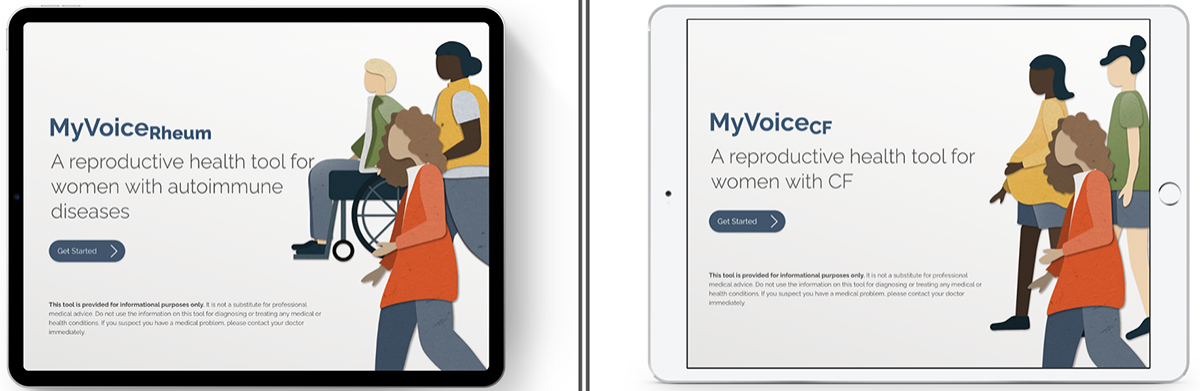
MyVoice:Rheum and MyVoice:CF tools for reproductive health decision-making for women with specific chronic illnesses.

We developed two decisions aids (see Figure 4)—MyVoice:Rheum and MyVoice:CF—to help women (1) conceptualize the benefits and potential health risks of parenting, pregnancy, and/or pregnancy avoidance in the context of their conditions; (2) recognize and refine their reproductive goals; and (3) communicate these goals to their health care teams. Tool development centered on engagement with patients, clinicians, and reproductive health specialists to ensure that the content was accurate (*Principle 1: Interdisciplinary stakeholder–inclusive teams*), reflected patients’ specific preferences and information priorities (*Principle 2: Supporting a patient-centered approach*), and helped to prepare patients for shared reproductive decision-making with clinicians in ways that enable them to receive the reproductive health care that meets their needs and preferences (*Principle 3: Advancing reproductive health equity*) [61,62]. Although the content of MyVoice:Rheum and MyVoice:CF is different and disease-specific, we developed the tools in tandem to maximize efficiencies related to time and expense.

We are currently undertaking feasibility testing for MyVoice:Rheum and MyVoice:CF.

## Avenues and Challenges to Ethical Dissemination

Our principles are, in theory, equally applicable to the design of femtech in both the academic and commercial spheres, and our own work straddles the spectrum from early prototypes to commercialization. However, the academic and commercial spheres operate on very different timelines and incentives. Academic research is, by nature, slow-moving. Its overarching goal is the creation of knowledge. The pace with which such knowledge is created is dependent on rigorous scientific design and is often at the mercy of federal or nonprofit funding cycles. Therefore, an idea is often formed many years before the work can even begin to be realized. The benefit of such a timeline is the luxury of a careful and scientifically grounded approach. However, the risk is not moving at a pace that is necessary for real-world impact. Commercial femtech, while often borne from academic research, can, and indeed must, move on a faster timeline, which can result in faster realization of a product that meets a pressing reproductive health need. The risk is prioritizing profit over patient-centeredness, especially in instances where the needs of patients and the desires of investors are not aligned, or if certain patients are not considered to constitute a profitable consumer base.

Within our own collaborative, we have tried different models. Most of us are currently evaluating our work with either federal or nonprofit funding (MyPath, MyDecision, MyVoice:Rheum, and MyVoice:CF). In contrast*,* MHP MyHealthyPregnancy has licensed intellectual property from the universities where it was built, leading to the development of a start-up company. The commercialization approach allows for continuity in delivering the product to the patient, preventing the need to cut off tool functionality due to a lapse in grant funding. For example, the MHP MyHealthyPregnancy platform was able to rapidly integrate triaging and education in response to the COVID-19 pandemic, which may not have been possible if relying solely on federal funding. However, forming a commercial entity may lead to the perception that the entity was created to monetize health disparities, regardless of how mission-driven product development is. As commercial products grow and scale, that skepticism can evolve into a true tension between social good and return on investment. Moreover, once a tool has moved out of the university setting, the researchers/developers no longer have access to an internal institutional review board to provide ethical oversight. This oversight may be particularly important for the protection of patients who are already socially or medically vulnerable. Several commercial femtech companies without such oversight have, without user consent, sold user data to third parties, including to the companies at which users work. Therefore, researchers wishing to offer sustainable and scalable services through commercialization must build the ethical imperative for responsible femtech into the driving mission of any commercial entity from the outset to ensure that the principles of equitable and high-quality care are upheld if and when transitioning from the academic to the market sphere.

Another important consideration for femtech dissemination is equitable outreach that extends beyond the research context. In each of our studies, we were able to proactively recruit diverse groups of participants, oftentimes partnering with community-based organizations or advocacy groups to do so. In some instances, we were able to provide phones or tablets for use by those with limited digital access. However, the question remains for how to ensure that these femtech tools continue to serve the needs and interests of those experiencing health, societal, and digital inequities once they are launched on a larger scale. While the principles proposed herein will ideally lead to high levels of engagement among populations that are typically underresourced, the technology still needs to get into the hands of those who may need it most. To achieve this, we advocate for a community-based partnership model of dissemination, including seeking consultation and partnership on continued product design and distribution with professional and nonprofit expert organizations. Women currently comprise the majority of Medicaid participants and Medicaid coverage is the highest among demographic groups experiencing the greatest health inequities [63]. Therefore, another promising dissemination pathway is state-based coverage, whereby individual states mandate the utilization of specific femtech innovations by Managed Care Organizations. Lastly, it is vital to make sure that the technology itself poses minimal access issues. In the United States, use of smartphones and similar digital devices is ubiquitous, yet the quality and consistency of digital service is not. For people living in rural areas or who have financial constraints around data plans, software development should ensure that the key features still work offline and on a variety of devices (eg, building in native code).

It is essential to take stock of what can and cannot be addressed by health technology. We believe that a limitation of emerging technology innovations is the conviction that technology presents an all-encompassing solution to societal ills. Experiences of societal, institutional, and interpersonal discrimination or marginalization require much more than a single technology-based solution. However, individuals facing health disparities cannot also be sacrificed to a digital divide, which may further potentiate health disparities. If digital technologies are developed by centering the unique needs of those who face the greatest health disparities, then femtech can begin to rectify some of the disproportionately experienced risk factors for poor outcomes. Without following these principles, even the most well-intentioned femtech risks further exacerbating inequities at the intersection of gender, race, and health.

## Looking Ahead

We recognize there will be missteps as we struggle to document truths and address gaps in care for underserved populations. We continue to explore the best ways to scale and disseminate our tools to reach those women with the greatest reproductive health disparities. We hope that others may contribute their own guiding principles and implementation strategies to the discussion and continue to refine our proposed approaches, so that the femtech community can aspire to enhance the health care quality and equity of all women.

## Abbreviations

ACOG: American College of Obstetricians and Gynecologists
CF: cystic fibrosis
IPV: Intimate Partner Violence
RMD: rheumatic and musculoskeletal disease
VA: Veterans Affairs
